# Spatial Constitutive Modeling of AA7050-T7451 with Anisotropic Stress Transformation

**DOI:** 10.3390/ma15175998

**Published:** 2022-08-30

**Authors:** Zhenda Wang, Xiuli Fu, Nianwei Xu, Yongzhi Pan, Yijia Zhang

**Affiliations:** Department of Mechanical Engineering, University of Jinan, Jinan 250022, China

**Keywords:** AA7050-T7451, anisotropy, coordinate transformation matrix, Johnson-Cook (JC) constitutive model, spatial constitutive model

## Abstract

The mechanical properties of anisotropic materials are generally characterized based on the orthotropy or transverse isotropy. However, the two-dimensional plane stress problems cannot comprehensively characterize the anisotropy of materials. In this study, based on the theory of elasticity and the transformation of the three-dimensional space coordinate system, combined with the projection relationship of the Cauchy stress tensor of an arbitrary section, the transformation relationship of the elastic modulus, shear modulus, and stress–strain between the orthogonal and load coordinate systems are obtained. The orthotropic Johnson-Cook (JC) constitutive model of AA7050-T7451 aluminum alloy is modified by fitting, and the constitutive relationship at any spatial angle is theoretically calculated by combining the obtained spatial coordinate transformation matrix. The generated spatial constitutive model is verified and modified through experiments. The results demonstrate that the theoretical mechanical properties and the modified spatial constitutive model can accurately reflect the effect of the spatial angle on the material stress distribution.

## 1. Introduction

Aluminum alloys are the most extensively used materials in industrial and civil applications and weapon manufacture, mainly due to their excellent properties, including high strength-to-weight ratio, low density, high-temperature resistance, and excellent corrosion resistance [1,2]. AA7050-T7451 aluminum alloy, which includes certain anisotropy because it is a typical poly crystalline alloy material, is typically used in aerospace applications for critical aircraft components [3,4,5]. Under different material pretreatment conditions, due to the different existence modes of micro-atoms (such as hydrogen) in the material, the grains in the material will produce different micro-effects, such as dislocation, twinning and other phenomena [6], so that the micro-grain of the material has complicated changes. Finally, the materials show different mechanical properties. Constitutive models have been effectively employed to characterize the mechanical properties of materials during plastic deformation [7,8]. Constitutive material modeling directly affects the accuracy of the theoretical study and finite element analyses [9]. Therefore, it is crucial to propose valid constitutive formulations that can describe the anisotropic characteristics for predicting the mechanical properties of materials.

The differences in the mechanical properties of anisotropic materials are generally manifested by the elastic modulus, Poisson’s ratio, and strength, in different directions [10,11,12]. Therefore, classical elastoplastic mechanics based on homogeneous, continuous, and isotropic assumptions cannot correctly reflect anisotropic mechanical behavior. The elastoplastic constitutive strength theory and the numerical analysis of anisotropic materials have been extensively researched. Plunkett et al. [13] proposed yield functions describing the anisotropic behavior of orthotropic sheet metal under tension and compression. Oana et al. [14] established a new isotropic criterion that well describes the yielding of randomly oriented face-centered polycrystalline metallic materials and extended this criterion to describe the orthotropy using generalized stress invariants. To describe the plastic anisotropy of metallic alloys, Cardoso et al. [15] developed a yield function that delivered an accurate prediction of directional stresses. Kazimierz et al. [16] studied a mathematical method for describing orthotropic material cutting based on the fracture theory and included the work of separation (fracture toughness) in addition to the material plasticity and friction. Meng et al. [17] established a modified JC constitutive model considering the forming direction, based on the dynamic impact shear test of AA7050-T7451 aluminum alloy in the typical forming direction (rolling direction (RD), transverse direction (TD), and normal direction (ND)).

The above studies were based on the orthotropic characteristics (RD, TD, ND) of materials because of the complexity of anisotropic mechanical behavior. In order to further study the anisotropy of materials, some scholars characterized the anisotropy of different orientations in a specific plane. Zhang et al. [18] accurately described the tensile stress–strain curves of anisotropic (along a certain angle to the rolling direction) aluminum alloy sheets using the three-parameter Barlat yield criterion. Aboubakr et al. [19] investigated the effect of the rolling orientation on the mechanical, in-plane anisotropy, and gamma ray shielding properties of Al-Cu-Li-Mg-X alloy. Based on the three-parameter Barlat yield criterion, Liu et al. [20] proposed a new five-parameter yield criteria in which two transverse shear stresses were added.

Although extensive research has been conducted on the anisotropy of materials, and yield criteria for describing their mechanical behavior have been proposed, the spatial conditions of orthotropy and transverse isotropy are yet to be resolved. The two-dimensional plane stress problem cannot comprehensively characterize the anisotropy of materials because three-dimensional characterization of the anisotropy cannot be realized.

Based on the theory of elasticity and the conversion of the space coordinate system, this study aims to obtain a theoretical method for comprehensively characterizing the anisotropy by introducing three-dimensional space angle variables. Quasi-static loading tests and dynamic impact compression/shear tests are performed on specimens in the typical forming direction (ND, TD, and RD) and space angle (RD-30°-ND, RD-45°-ND, RD-60°-ND). The quasi-static and high-strain-rate mechanical responses at different rates, directions, and temperatures are analyzed, and the proposed theoretical method is verified. Finally, the least squares method is used for fitting and modifying the constitutive material parameters, and a JC constitutive model that includes the spatial angle is established by combining the coordinate transformation matrix. The modified material constitutive model will be applied to the theoretical research and finite element simulation of material mechanical properties.

## 2. Theoretical Research on Anisotropy

### 2.1. Coordinate-System Conversion Matrix

The main form of metal plastic deformation is the crystal slip, which proceeds along a certain crystal plane and direction [21]. The strongest interatomic binding force has the least probability of disorder in the arrangement of atoms; hence, crystal slip preferentially occurs in the crystal plane with the highest atomic density and the crystal orientation with the densest atomic arrangement [22]. According to plastic mechanics, until the stress acting on the slip surface and direction reaches the critical value, the slip will proceed along a specific direction. AA7050-T7451 aluminum alloy is a typical face-centered cubic lattice, which consists of four groups of preferred slip surfaces [23,24,25,26]. As there are three preferred sliding directions for each slip surface, AA7050-T7451 has 12 slip systems and 384 slip states [27,28]. Based on the above analysis, it can be concluded that the simple two-dimensional plane stress problem cannot accurately characterize the anisotropy. In order to comprehensively characterize material anisotropy, a constitutive model that can characterize the mechanical parameters in each direction of the three-dimensional space must be established.

**Figure 1:**
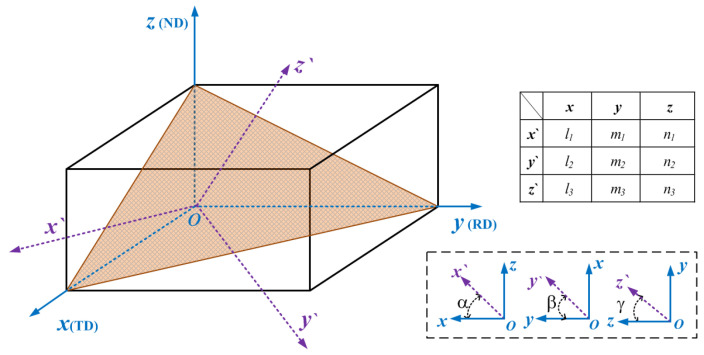
Positional relationship and directional cosine between the axes of the orthogonal and load coordinate systems.

In the mechanical properties of anisotropic materials, the differences are mainly manifested by the elastic modulus, shear modulus, and stress. When anisotropic materials are subjected to non-orthotropic loads, the assumptions of orthotropy and transverse isotropy can no longer satisfy the descriptions of their stress parameters. Therefore, a load coordinate system must be established to characterize the mechanical parameters, under the load direction. An orthogonal (material) coordinate system was established with the *x*, *y*, and *z* axes of the TD, RD, and ND, respectively. A load coordinate system was created by considering the load direction as the *z*’ axis; thus, the three axes of it are not along the elastic direction (TD, RD and ND). The positional relationship between the axes of the orthogonal and load coordinate systems is shown in Figure 1; *α*, *β*, and *γ* represent the angle between the *x*, *y*, and *z* axes of the load coordinate system (*ox*′*y*′*z*′) and orthogonal coordinate system (*oxyz*), respectively, while *l_i_*, *m_i_*, and *n_i_* represent the directional cosine between the coordinate axes. To characterize the mechanical properties in any direction of the three-dimensional space, the transformation relationship between the load and material coordinate systems must be obtained, and the orthotropic mechanical parameters in the material coordinate system and coordinate transformation matrix must be combined [29].

**Figure 2:**
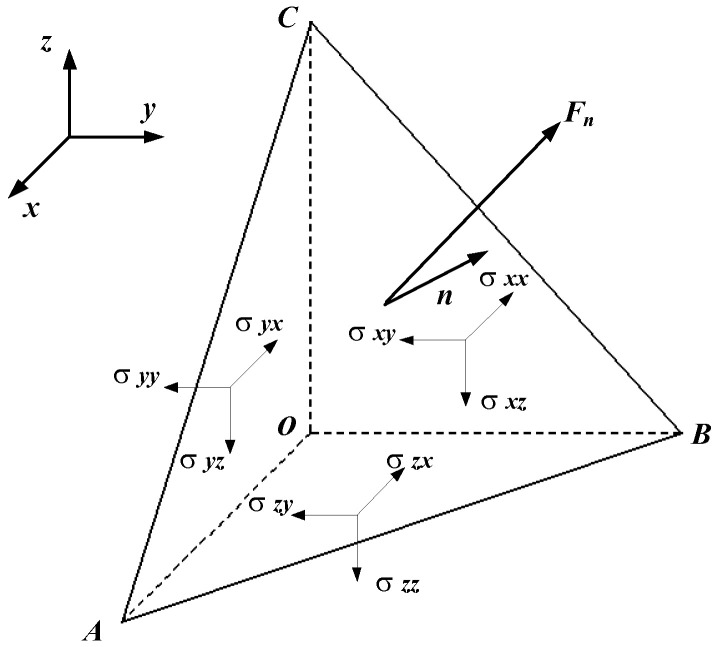
Stress component of an arbitrary elastomer section.

According to the theory of elasticity, the stress vector *F_n_* of any oblique section can be uniquely determined by nine stress components in the elastic body, also known as the Cauchy stress tensor [30]. Figure 2 illustrates the stress components (positive stress components *σ_xx_*, *σ_yy_* and *σ_zz_*, and the shear stress components) of the Cauchy tensor that completely describe the stress state at an arbitrary point in an elastic body. The normal stress component represents the tensile or compressive stress on the corresponding section. As per the equivalent law of the shearing stress, the shear stress can be expressed by *τ_xy_*, *τ_yz_* and *τ_zx_* [31,32]. The expression for the stress tensor σ is as follows:

(1) $$\sigma =\left[\begin{array}{ccc} \sigma_{xx} & \sigma_{xy} & \sigma_{xz}\\ \sigma_{yx} & \sigma_{yy} & \sigma_{yz}\\ \sigma_{zx} & \sigma_{zy} & \sigma_{zz} \end{array}\right]=\left[\begin{array}{ccc} \sigma_{xx} & \tau_{xy} & \tau_{zx}\\ \tau_{xy} & \sigma_{yy} & \tau_{yz}\\ \tau_{zx} & \tau_{yz} & \sigma_{zz} \end{array}\right]$$

Therefore, the stress state of any cross-section can be characterized by the stress parameters in the orthogonal state. The intermediate variable is given by the positional relationship between the cross-section and orthogonal coordinate system, i.e., the transformation relationship between the load and orthogonal coordinate systems. Using the above transformation matrix, the stress parameters obtained in the orthogonal state can be transformed into the stress state of an arbitrary cross-section, which can be used to characterize the mechanical parameters in three-dimensional space [33,34,35].

Considering the normal direction of an oblique section *ABC* as the *z*′ axis of the new coordinate system, the stress components *X_v_*, *Y_v_*, and *Z_v_* on the section along the original coordinate axis can be expressed as follows:

(2) $$\begin{array}{l} X_{v}=\sigma_{x}l_{3}+\tau_{xy}m_{3}+\tau_{xz}n_{3}\\ Y_{v}=\tau_{yx}+\sigma_{y}m_{3}+\tau_{yz}n_{3}\\ Z_{v}=\tau_{zx}l_{3}+\tau_{zy}m_{3}+\sigma_{z}n_{3} \end{array}$$

Projecting *X_v_*, *Y_v_*, and *Z_v_* onto the *z*′ axis, the normal stress $\sigma_{z^{\prime }}$ of *ABC* can be obtained as follows:

(3) $$\sigma_{z^{\prime }}=X_{v}l_{3}+Y_{v}m_{3}+Z_{v}n_{3}=\sigma_{x}l_{3}{}^{2}+\sigma_{y}m_{3}{}^{2}+\sigma_{z}n_{3}{}^{2}+2\tau_{xy}l_{3}m_{3}+2\tau_{yz}m_{3}n_{3}+2\tau_{zx}n_{3}l_{3}$$

When the tetrahedral differential body *OABC* tends to be infinitely small, the *x*′ and *y*′ axes are in the oblique section, *ABC.* Therefore, the projection of *X_v_*, *Y_v_,* and *Z_v_* on the *x*′ and *y*′ axes indicates the shear stress of the oblique differential body section.

(4) $$\tau_{x^{\prime }z^{\prime }}=\tau_{z^{\prime }x^{\prime }}=X_{v}l_{1}+Y_{v}m_{1}+Z_{v}n_{1}=\sigma_{x}l_{1}l_{3}+\sigma_{y}m_{1}m_{3}+\sigma_{z}n_{1}n_{3}+\tau_{xy}\left(l_{3}m_{1}+l_{1}m_{3}\right)+\tau_{yz}\left(m_{1}n_{3}+m_{3}n_{1}\right)+\tau_{zx}\left(l_{1}n_{3}+l_{3}n_{1}\right)$$

(5) $$\tau_{y^{\prime }z^{\prime }}=\tau_{z^{\prime }y^{\prime }}=X_{v}l_{12}+Y_{v}m_{2}+Z_{v}n_{2}=\sigma_{x}l_{2}l_{3}+\sigma_{y}m_{2}m_{3}+\sigma_{z}n_{2}n_{3}+\tau_{xy}\left(l_{2}m_{3}+l_{3}m_{2}\right)+\tau_{yz}\left(m_{2}n_{3}+m_{3}n_{2}\right)+\tau_{zx}\left(l_{2}n_{3}+l_{3}n_{2}\right)$$

The stress tensor of the oblique section, *ABC*, in the new coordinate system, *ox*′*y*′*z*′, can be expressed as

(6) $$\left[\sigma^{\prime }\right]=\left[\begin{array}{ccc} \sigma_{x^{\prime }} & \tau_{x^{\prime }y^{\prime }} & \tau_{x^{\prime }z^{\prime }}\\ \tau_{y^{\prime }x^{\prime }} & \sigma_{y^{\prime }} & \tau_{y^{\prime }z^{\prime }}\\ \tau_{z^{\prime }x^{\prime }} & \tau_{z^{\prime }y^{\prime }} & \sigma_{z^{\prime }} \end{array}\right]$$

The transformation matrix of the stress tensor from the original coordinate system *oxyz* to the new coordinate system *ox*′*y*′*z*′ can be obtained by combining Equations (2)–(6).

(7) $$\left[\sigma^{\prime }\right]=\left[A\right]\left[\sigma \right]{\left[A\right]}^{T}$$

where [*A*] = $\left[\begin{array}{ccc} l_{1} & m_{1} & n_{1}\\ l_{2} & m_{2} & n_{2}\\ l_{3} & m_{3} & n_{3} \end{array}\right]$ is the coordinate transformation matrix.

Therefore, the stress in the load coordinate system can be obtained by combining the mechanical parameters of the orthogonal coordinate system and the coordinate transformation matrix. Thus, the breakthrough from orthotropy to anisotropy characterization in three-dimensional space can be realized.

### 2.2. Mechanical Properties of the Anisotropy

The transformation of the stress state between the coordinate systems can be realized using the transformation matrix between the load and the orthogonal coordinate systems. As previously mentioned, the difference in the mechanical characteristics of anisotropic materials is mainly manifested by the elastic modulus, shear modulus, and stress–strain parameters; therefore, the transformation relationships of the strain, elastic modulus, and shear modulus between the orthogonal coordinate system and load coordinate system can be further analyzed using the transformation matrix.

In the material coordinate system *oxyz*, the stress–strain relationship of anisotropic materials can be expressed as follows:

(8) $$\left[\varepsilon \right]=\left[S\right]\left[\sigma \right]$$

According to the theory of elasticity and the transformation relationship of the space coordinate system, the stress–strain relationship in the load coordinate system can be obtained as follows:

(9) $$\left[\varepsilon^{\prime }\right]=\left[T\right]\left[S\right]\left[\sigma \right]=\left[T\right]\left[S\right]{\left[T\right]}^{T}\left[\sigma^{\prime }\right]=\left[S^{\prime }\right]\left[\sigma^{\prime }\right]$$

where $\;\left[\varepsilon^{\prime }\right]\;$ is the strain vector in the load coordinate system, $\left[T\right]$ is the flexibility conversion matrix, $\left[S\right]$ is the anisotropic flexibility matrix, $\left[\sigma \right]\;$ is the stress vector in the material coordinate system, $\left[\sigma^{\prime }\right]\;$ is the stress vector in the load coordinate system, $\left[S^{\prime }\right]=\left[T\right]\left[S\right]{\left[T\right]}^{T}$ is the equivalent anisotropic flexibility matrix in the load coordinate system, $\left[T\right]$ and $\;\left[S\right]$ are expressed by Equations (10) and (11), respectively.

(10) $$\left[T\right]\;=\;\left[\begin{array}{cccccc} l_{1}{}^{2} & m_{1}{}^{2} & n_{1}{}^{2} & l_{1}m_{1} & m_{1}n_{1} & n_{1}l_{1}\\ l_{2}{}^{2} & m_{2}{}^{2} & n_{2}{}^{2} & l_{2}m_{2} & m_{2}n_{2} & n_{2}l_{2}\\ l_{3}{}^{2} & m_{3}{}^{2} & n_{3}{}^{2} & l_{3}m_{3} & m_{3}n_{3} & n_{3}l_{3}\\ 2l_{1}l_{2} & 2m_{1}m_{2} & 2n_{1}n_{2} & l_{1}m_{2}\;+\;l_{2}m_{1} & m_{1}n_{2}\;+\;m_{2}n_{1} & n_{1}l_{2}\;+\;n_{2}l_{1}\\ 2l_{2}l_{3} & 2m_{2}m_{3} & 2n_{2}n_{3} & l_{2}m_{3}\;+\;l_{3}m_{2} & m_{2}n_{3}\;+\;m_{3}n_{2} & n_{2}l_{3}\;+\;n_{3}l_{2}\\ 2l_{2}l_{3} & 2m_{3}m_{1} & 2n_{3}n_{1} & l_{3}m_{1}\;+\;l_{1}m_{3} & m_{3}n_{1}\;+\;m_{1}n_{3} & n_{3}l_{1}\;+\;n_{1}l_{3} \end{array}\right]$$

(11) $$\left[S\right]=\left[\begin{array}{cccccc} s_{11} & s_{12} & s_{13} & s_{14} & s_{15} & s_{16}\\ & s_{22} & s_{23} & s_{24} & s_{25} & s_{26}\\ & & s_{33} & s_{34} & s_{35} & s_{36}\\ & & & s_{44} & s_{45} & s_{46}\\ & & & & s_{55} & s_{56}\\ & & & & & s_{66} \end{array}\right]$$

The elastic modulus *E* and shear elastic modulus *G* of the tetragonal system in an arbitrary direction can be obtained as follows [36]:

(12) $$E^{-1}=\left(R_{1}{}^{4}+R_{2}{}^{4}\right)S_{11}+R_{3}{}^{4}S_{33}+\left(R_{1}{}^{2}+R_{2}{}^{4}\right)R_{3}{}^{2}\left(2S_{13}+S_{44}\right)+R_{1}{}^{2}R_{2}{}^{2}\left(2S_{12}+S_{66}\right)$$

(13) $$G^{-1}=2S_{11}\left[R_{1}\left(1-R_{1}{}^{2}\right)+R_{2}{}^{2}\left(1-R_{2}{}^{2}\right)\right]+2S_{33}R_{3}{}^{2}\left(1-R_{3}{}^{2}\right)-4S_{12}R_{1}{}^{2}R_{2}{}^{2}-4S_{13}\left(R_{1}{}^{2}R_{3}{}^{2}+R_{2}{}^{2}R_{3}{}^{2}\right)\;+\frac{1}{2}S_{44}\left(2-R_{1}{}^{2}-4R_{2}{}^{2}R_{3}{}^{2}-R_{2}{}^{2}-4R_{1}{}^{2}R_{3}{}^{2}\right)+\frac{1}{2}S_{66}\left(1-R_{3}{}^{2}-4R_{1}{}^{2}R_{2}{}^{2}\right)$$

where *R_i_* is the direction cosine of the load coordinate system relative to the orthogonal coordinate system, and *S_ij_* is the flexibility coefficient of the material.

The experimental data (elastic modulus, Poisson’s ratio, shear modulus, and stress–strain) in different directions of anisotropic material specimens were obtained through correlative experiments. The theoretical values corresponding to the load coordinate system, which can be experimentally verified, were obtained by combining the transformation matrix. Subsequently, the constitutive equation that can comprehensively characterize the anisotropic mechanical characteristics was stablished and corrected.

## 3. Experimental

### 3.1. Specimen Preparation

**Figure 3:**
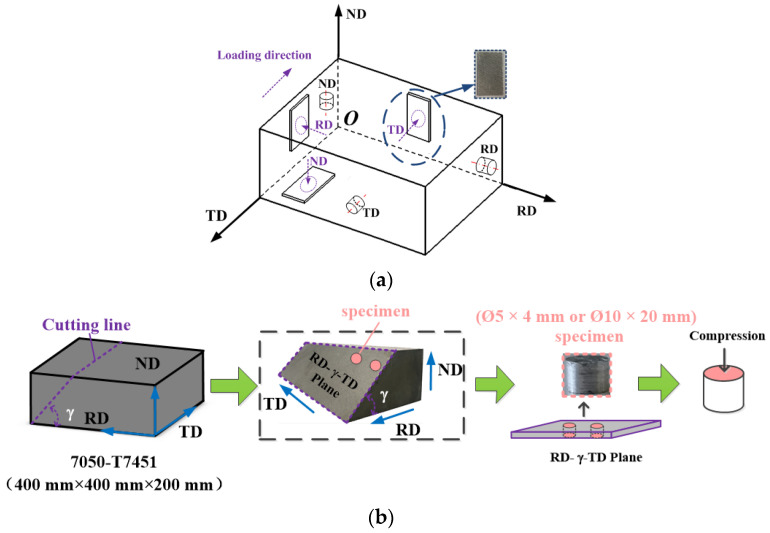
(**a**) Sampling in the typical forming direction. (**b**) Sampling at different forming angles (*γ* = 0°, 30°, 45°, 60°, 90°).

Pre-stretched AA7050-T7451 sheets were used in this study. In order to obtain the mechanical parameters of the material coordinate system, the specimens were sampled along the typical forming directions (RD, TD, and ND) of the sheet. A specific angle plane was selected for sampling to verify the theoretically derived results; the sampling direction is depicted in Figure 3. In Figure 3a, the specimen directions for impact compression and shear are respectively defined along their loading directions, namely, the axis direction of the cylinder and the thickness direction of the shear specimen. As shown in Figure 3b, the angle plane was selected based on the TD-RD plane at a certain angle *γ* (*γ* = 0°, 30°, 45°, 60°, 90°), which was defined as the RD-*γ*-TD plane. Cylindrical specimens were processed by wire-cutting in the RD-*γ*-TD plane; specimens at 0° and 90° constituted the ND and RD samples, respectively. Quasi-static compression tests were carried out at 25 °C (room temperature), and specimens sized Ø10 × 20 mm were sampled in the RD, TD, and ND, respectively. Impact compression and impact shear tests were performed using samples sized Ø5 × 4 mm and 40 × 23.5 ×2 mm (L × D × H), respectively.

### 3.2. Experimental Setup and Principle

**Figure 4:**
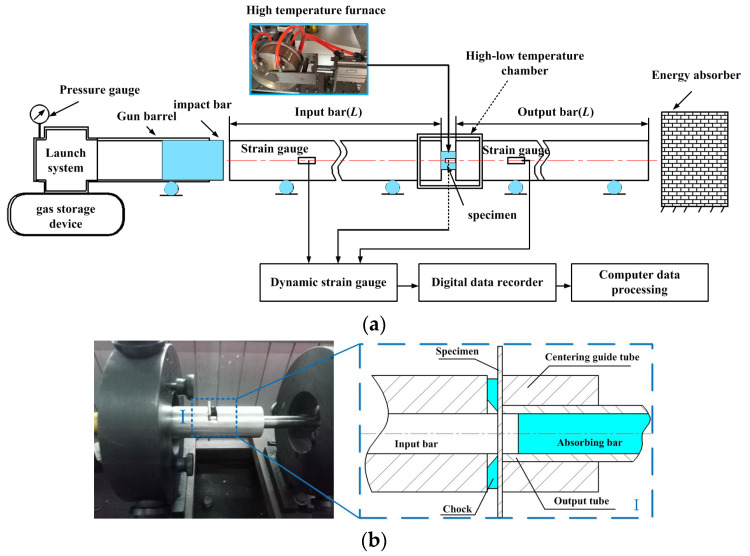
(**a**) SHPB device. (**b**) Dynamic “Bar-Tube” shearing device.

Dynamic impact compression and shear tests were performed using a split Hopkinson pressure bar (SHPB) device, which is a typical method for obtaining the dynamic mechanical properties [37]. The elastic incident and reflected waves were measured by an input bar strain gauge. Further, the elastic incident wave was measured by an output bar strain gauge and was amplified by a dynamic strain gauge. The waveform data were collected and stored using a digital data recorder. Finally, the dynamic–mechanical property data were obtained according to the one-dimensional stress wave theory [38,39]. As the ultrahigh strain rate in the actual cutting process cannot be achieved by the SHPB device during the impact shearing process, a dynamic impact shearing “bar-tube” device was used to enhance the SHPB. The SHPB test equipment and “bar-tube” device are depicted in Figure 4. An intelligent temperature-controlled electric heating furnace was used for heating the specimens at a maximum temperature of 800 °C and temperature control precision of ±1 °C.

At room temperature (25 °C), quasistatic compression tests were carried out on specimens (Ø10 × 20 mm) in different directions (RD, ND, TD) at a strain rate of 1.6 × 10^−3^ s^−1^. Dynamic impact shear tests at strain rates of 1.0 × 10^4^, 2.0 × 10^4^, and 3.0 × 10^4^ s^−1^ were conducted on shear specimens with different orientations (RD, ND, TD). Further, dynamic impact compression tests were performed on cylindrical specimens (Ø5 × 4 mm) in different directions (RD, ND, TD, and RD-*γ*-TD) at strain rates of 0.1 × 10^4^, 0.2 × 10^4^, 0.4 × 10^4^, and 0.6 × 10^4^ s^−1^, respectively. In order to obtain the mechanical properties at high temperature, impact compression tests were conducted at a strain rate of 0.4 × 10^4^ s^−1^, at 100, 200, 300, 350, 400, and 450 °C, respectively. Each group of experiments was repeated thrice, and the experimental results were averaged to ensure accuracy.

## 4. Results and Discussion

### 4.1. Establishment of a Anisotropic Constitutive Model

**Figure 5:**
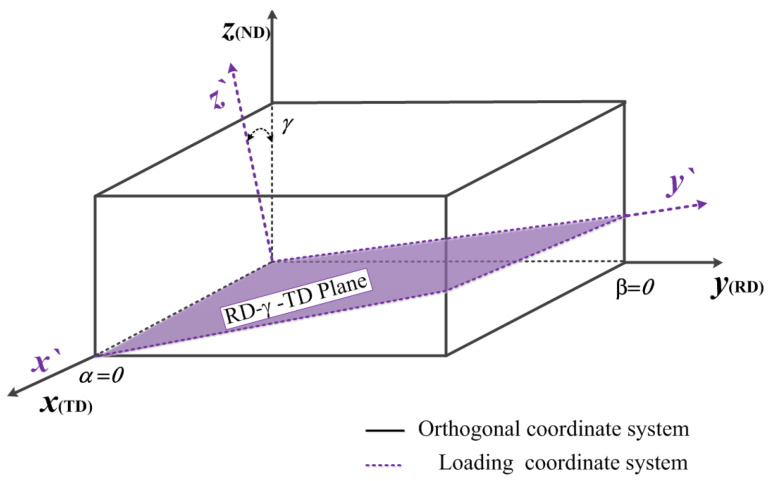
Relationship between the orthogonal and load coordinate systems of AA7050-T7451.

As displayed in Figure 5, based on the selection of the angle plane in this study, the load coordinate system under angle *γ* was established as per Figure 1 and Figure 3b. According to the positional relationship between the orthogonal and load coordinate systems, the cosine of each direction was obtained and substituted into the coordinate system transformation matrix [*A*]. As depicted in Equation (14), the stress relationship expression between the load and orthogonal coordinate systems can be obtained by substituting the transformation matrix [*A*] of the obtained coordinate system in Equation (7).

(14) $$\left[\sigma^{\prime }\right]=\left[\begin{array}{ccc} \sigma_{xx} & \tau_{xy}cos\gamma & \tau_{xy}sin\gamma +\tau_{xy}cos\gamma \\ \tau_{yx}cos\gamma & \sigma_{yy}{cos}^{2}\gamma & \sigma_{y}sin\gamma cos\gamma +\tau_{yz}cos\gamma \\ \tau_{yx}sin\gamma +\tau_{xz}cos\gamma & \left(\sigma_{yy}+\tau_{yz}cos\gamma \right)cos\gamma & \sigma_{yy}sin^{2}\gamma +\sigma_{zz}{cos}^{2}\gamma +2\tau_{zy}cos\gamma sin\gamma \end{array}\right]$$

The normal stress at 0° (ND) and 90° (RD) is *σ_zz_* and *σ_yy_*, respectively, in the above equation. The stress in the orthogonal coordinate system is directly related to the accuracy of the theoretical stress value in the load coordinate system. Therefore, the constitutive equation in the orthogonal coordinate system, which can accurately express the stress–strain relationship of each direction in this system, must be established and modified.

The constitutive equation of a material describes the stress–strain relationship of the material under different loading conditions [40,41]. The empirical Johnson–Cook constitutive model was applied to describe the stress–strain relationship of ferrous and nonferrous metals, under conditions of high-strain rate and considerable deformation. In addition, the structure of the JC constitutive model is simple and convenient; it introduces the strain strengthening, strain-rate strengthening, and thermal softening parameters of the material [42], and can be expressed as follows:

(15) $$\sigma =\left(A+B\varepsilon^{n}\right)\left(1+C\ln\dot{\varepsilon }\right)\left[1-{\left(\frac{T-T_{r}}{T_{m}-T_{r}}\right)}^{m}\right]$$

where *A*, *B*, *C*, *n*, and *m* represent the initial yield stress, strain hardening modulus, hardening index, strain-rate sensitivity coefficient, and thermal softening coefficient, respectively; *T*, *T_r_*, and *T_m_* represent the deformation temperature, room temperature, and melting temperature of the material, respectively; *σ*, *ε*, and $\dot{\varepsilon }$ are the stress, strain, and reference strain rate, respectively [43].

For anisotropic materials, the constitutive model should be constructed in the typical forming direction and fitted in the orthogonal coordinate system. If the modified JC constitutive model can accurately characterize the stress–strain relationship of the material in the orthogonal coordinate system, the stress–strain constitutive relationship in the load coordinate system at an arbitrary angle *γ* can be obtained by combining Equations (14) and (15):

(16) $$\sigma_{\gamma }=\left(\sigma_{RD}sin^{2}\gamma +\sigma_{ND}cos^{2}\gamma +2\tau cos\gamma sin\gamma \right)$$

### 4.2. Anisotropic Mechanical Property Analysis of AA7075-T7451

**Figure 6:**
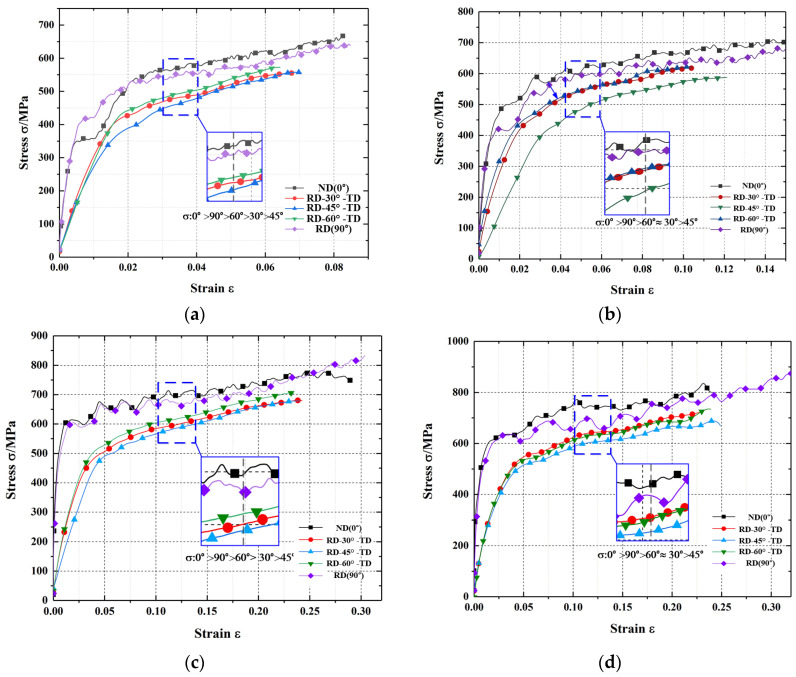
Stress–strain curves of AA7050-T7451 in different directions. (**a**) 0.1 × 10^4^ s^−^^1^; (**b**) 0.2 × 10^4^ s^−^^1^; (**c**) 0.4 × 10^4^ s^−^^1^; (**d**) 0.6 × 10^4^ s^−^^1^.

In order to modify the constitutive model and verify the anisotropy in three-dimensional space, dynamic impact compression tests were performed at different strain rates and temperatures for the specimens, in different directions. The stress–strain curves of the dynamic impact compression tests in the 0.1 × 10^4^–0.6 × 10^4^ s^−1^ strain-rate range were obtained, as depicted in Figure 6. When the strain was less than 0.02, the material was in the elastic deformation stage, and the stress increased rapidly because of the minor deformation caused by work hardening. With the increase in strain, thermal softening effect occurred in the material, and along with work hardening caused a significant decrease in the stress rate. When the work hardening and thermal softening effect gradually attained equilibrium, the stress exhibited a stable change stage. With the increase in strain rate, the maximum corresponding strain increased from 0.08 to 0.35. Meanwhile, the maximum stress in the stable stage also exhibited an upward trend, from 653 to 847 MPa.

At the same strain rate, the stresses at different angles *γ*, differed in the elastic, plastic deformation, and stable stages. As the material stress fluctuation was less in the stable deformation stage, the stress corresponding to the initial strain in the stable stage was selected [44]. The influence of angle *γ* on the stress was analyzed by comparing the stability stress under different loading conditions. Comparing the stability stresses at different strain rates, the variation trend of the stress with angle *γ* was as follows: The stress decreased with the increase in angle *γ* and reached a minimum when *γ* = 45°. As *γ* continued to increase, the stress increased after reaching a minimum value. Therefore, in the 0–90° range, the stress initially decreased and then increased with *γ*.

**Figure 7:**
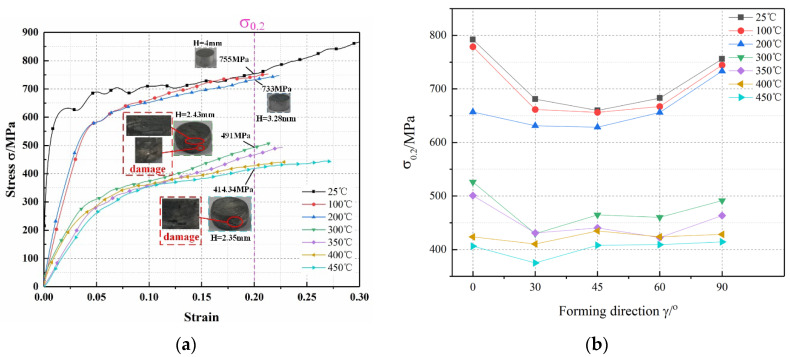
High-temperature mechanical characteristic curve of AA7050-T7451 (strain rate: 0.4 × 10^4^ s^−^^1^). (**a**) Stress–strain curves at different temperatures (RD). (**b**) Variation in the stability stress with *γ* at different temperatures.

Figure 7 illustrates the stress–strain curves of AA7050-T7451 at different temperatures, for a strain rate of 0.4 × 10^4^ s^−1^. When the temperature increased from room temperature to 200 °C, the height of the specimen decreased by only 0.72 mm, and the stress decreased from 755 to 733 MPa; therefore, the effect of the temperature on the stress of the material was less when the temperature was below 200 °C. When the temperature reached 300 °C, the stress decreased sharply, and the flow stress was only 60–70% of that at 200 °C. The specimen height decreased by 0.85 mm, compared to that at 200 °C, and the specimens exhibited obvious damage. The thermal activation increases the atomic kinetic energy in the alloy, when the deformation temperature is increased to a certain value, resulting the rapid softening. Further, the critical shear stress of the alloy slip systems decreases, and the dislocation slip can easily overcome the hindrance effect at lower stress [45]. Therefore, the flow stress of AA7050-T7451 decreases with the increase in temperature.

Different from the mechanical curves under different strain rates, the initial strain of the stable deformation stage at different temperatures was approximately 0.2; therefore, the stress value at a strain of 0.2 was selected as the stability stress (*σ*_0_._2_) to analyze the variation trend, under different deformation temperatures. From Figure 7b, it can be observed that the variation in stress with temperature is consistent in the RD direction. When the temperature was below 300 °C, the stress of 0–90° specimens initially decreased and then increased, at the same temperature. The stress of the 45° specimen was the least, consistent with that at room temperature. Thus, the steady-state stress in each direction exhibited obvious anisotropy during the dominant stage of work hardening. Loading the materials at angles of 30°, 45°, and 60° reduced the stress, particularly, the minimum stress required for the 45° specimen deformation. The main reason is because the grain slip system in the material is anisotropic, and the preferred slip system direction is that with the highest atomic density or densest atomic arrangement. Therefore, it can be concluded that AA7050-T7451 material has the densest atomic arrangement in the direction of 45°, resulting in preferential slip orientation in this direction. The thermal softening effect was dominant at temperatures beyond 300 °C. With the increase in deformation temperature, the kinetic energy of the atoms in the material increased, and the crystal slip directions were no longer obvious.

In conclusion, the stress of AA7050-T7451 material is not only related to the strain rate, strain, and temperature, but also to the angle of its forming direction. Therefore, it is necessary to construct and modify the constitutive equation to include the spatial angle function, for describing the mechanical properties of the material.

### 4.3. Modification of the Constitutive Model

#### 4.3.1. Constitutive Model in the Orthogonal Coordinate System

In order to introduce the spatial angle function and obtain the constitutive model of the anisotropy in three-dimensional space, the stress–strain curves in the RD, TD, and ND were initially fitted and modified. The constitutive equation that can accurately characterize the stress relationship in the orthogonal coordinate system was obtained. The parameters of the JC model *A*, *B*, *C*, and *n* were fitted and modified using the experimental stress–strain curve along with least squares method, as shown in Table 1.

As the single thermal softening coefficient m cannot satisfy the mechanical characteristic curve of AA7050-T7451 at high temperature, the thermal softening effect term *K_t_* was polynomial fitted for accurately describing the rheological properties of the material at high temperature [46].

(17) $$K_{t}=\sigma /\left(A+B\varepsilon^{n}\right)\left(1+C\ln\dot{\varepsilon }\right)$$

**Figure 8:**
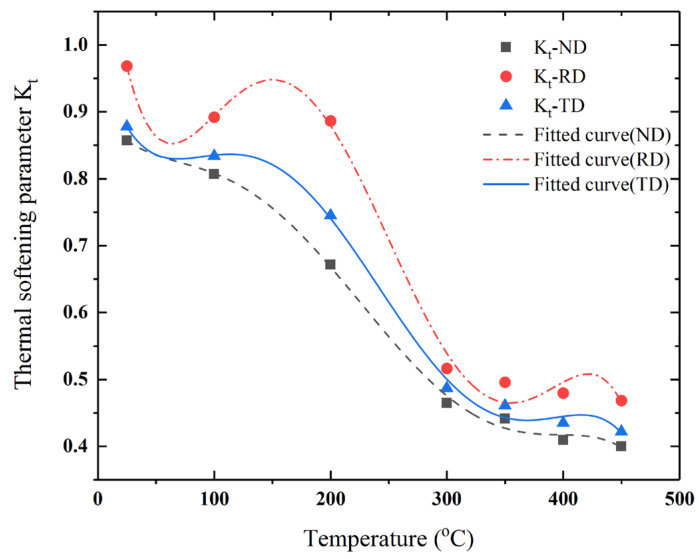
Fitting curves of the thermal softening coefficient for different directions with a strain rate of 0.4 × 10^4^ s^−^^1^.

Using the stress–strain curve of the high-temperature impact compression test, *K_t_* at different temperatures can be obtained, i.e., *K_t_* can be calculated by substituting the stress with a strain rate of 0.4 × 10^4^ s^−1^ and strain of 0.1 at different temperatures in Equation (16). If the number of polynomials is high, the expression will be complicated, and if the number of times is less, the fitting effect will be poor. Therefore, fifth-degree polynomial fitting with a simple expression and good fitting effect was used to fit the result, as shown in Figure 8. When the temperature was below 300 °C, the thermal softening coefficient decreased gradually with the increase in temperature, decreasing by approximately 0.13 only; in addition, the thermal softening coefficients in the TD, RD, and ND directions were considerably different. Sharp softening occurred at 300 °C, and the thermal softening coefficient dropped sharply to approximately 0.45; although the thermal softening coefficients in all the directions were different, the difference was not significant. After 300 °C, the thermal softening coefficient exhibited a small downward trend with the change in temperature, decreasing by 0.05 only, for a temperature increase of approximately 150 °C. Thus, the fitting curves can better reflect the mechanical properties of AA7050-T7451 material at high temperature.

Therefore, by fitting and modifying the parameters of the JC constitutive model, a constitutive equation that can accurately describe the mechanical properties of AA7050-T7451 was obtained, under different strain rates, temperatures, and forming directions (ND, RD, and TD):

(18) $$\left\{\begin{array}{l} \sigma_{ND}=\left(365+516\varepsilon^{0.27}\right)\left(1+0.042\ln\dot{\varepsilon }\right)K_{t-ND}\\ \sigma_{RD}=\left(342+452\varepsilon^{0.26}\right)\left(1+0.029\ln\dot{\varepsilon }\right)K_{t-RD}\\ \sigma_{TD}=\left(357+490\varepsilon^{0.3}\right)\left(1+0.04\ln\dot{\varepsilon }\right)K_{t-TD} \end{array}\right.$$

(19) $$K_{t}=\left\{\begin{array}{c} ND:0.9-0.002t+3.23\times 10^{-5}t^{2}-2\times 10^{-7}t^{3}+5.9\times 10^{-10}t^{4}-5.1\times 10^{-13}t^{5}\\ RD:1.2-0.016t+2.22\times 10^{-4}t^{2}-1.22\times 10^{-6}t^{3}+2.8\times 10^{-9}t^{4}-2.3\times 10^{-12}t^{5}\\ TD:0.98-0.006t+9.13\times 10^{-5}t^{2}-5.43\times 10^{-7}t^{3}+1.29\times 10^{-10}t^{4}-1.07\times 10^{-13}t^{5} \end{array}\right.$$

#### 4.3.2. Verification of the Elastic Modulus and Shear Modulus

In order to verify the feasibility of the theoretical method, the elastic modulus, shear modulus, and Poisson’s ratio of the typical forming direction (orthogonal coordinate system) were obtained, through the dynamic impact compression and shear tests. The angles between the orthogonal and load coordinate systems were *α* = 0, *β* = 0, and *γ*. The anisotropic flexibility matrix of AA7050-T7451 aluminum alloy material was calculated as follows:

(20) $$\left[S\right]=\left[\begin{array}{cccccc} 0.0135 & -0.0128 & -0.0106 & & & \\ -0.0152 & 0.0143 & -0.01187 & & & \\ -0.0171 & -0.0162 & 0.0134 & & & \\ & & & 0.04115 & & \\ & & & & 0.0455 & \\ & & & & & 0.0446 \end{array}\right]$$

**Figure 9:**
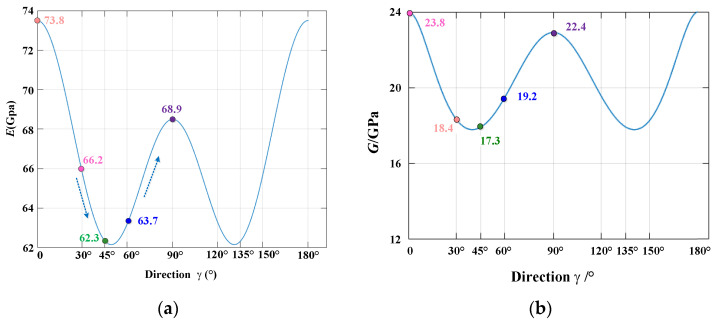
Elastic modulus and shear modulus with the change in *γ*: (**a**) elastic modulus, (**b**) shear modulus.

According to the flexibility matrix [*S*] of AA7050-T7451 and combining Equations (12) and (13), the change trends of the theoretical values of the elastic modulus and shear modulus with the change in *γ* were obtained, as shown in Figure 9. As *γ* increased, the theoretical elastic modulus and shear modulus values initially decreased and then increased, and the minimum value appeared at 45°. This trend was approximately the same as that of the stress change in the impact compression test, shown in Figure 6 and Figure 7. The change trends of the theoretical values of the elastic modulus and shear modulus were the same as that of the experimental values, verifying the accuracy of the theory and the method for characterizing the three-dimensional space anisotropy.

#### 4.3.3. Modification of the Spatial Constitutive Model

**Figure 10:**
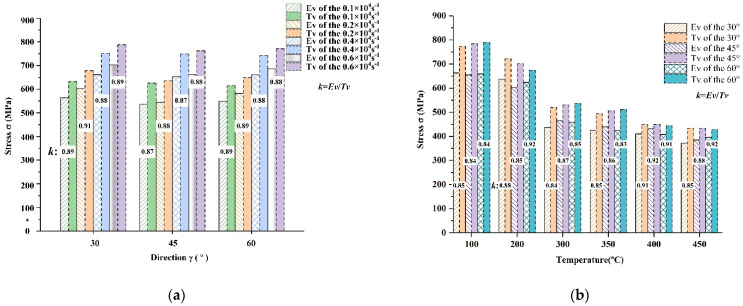
Comparison and verification of the theoretical and experimental values of the stress in the load coordinate system. (**a**) Test verification at different strain rates. (**b**) Test verification at different temperatures.

By comparing the experimental and theoretical values of the elastic modulus and shear modulus, respectively, the accuracy of the theoretical method for characterizing the three-dimensional mechanical parameters was preliminarily verified. To completely characterize the anisotropy of materials in three dimensions, the stress state should be characterized and verified to establish a constitutive relationship containing the spatial angle. In order to further verify the accuracy of the theoretical method, the theoretical value was verified using the results of the dynamic impact compression test. The stress obtained by dynamic impact compression is the normal stress ${\sigma_{z}}^{\prime }$, in the load coordinate system. The stresses in the stable stage depicted in the stress–strain curves at different strain rates in different directions were selected and verified. The verification results are presented in Figure 10.

In Figure 10, the verification results demonstrate that under different strain rates and temperature conditions, the change trend of the obtained theoretical values under different loading conditions is consistent with the test results. A certain gap existed between the theoretical value of the stress and the experimental value at various angles. Although the error was large, the ratio between the theoretical and experimental values was within a range of 0.85–0.90 because the theoretically calculated stress values did not consider the effects of the material preforming processes on the anisotropy. Therefore, a correction factor *k* needs to be included, which accurately characterizes the mechanical properties of the anisotropy of AA7050-T7451 aluminum alloy in three-dimensional space. The final expression for the stress–strain constitutive equation in the load coordinate system as follows:

(21) $$\sigma_{\gamma }=k\left(\sigma_{RD}{sin}^{2}\gamma +\sigma_{ND}{cos}^{2}\gamma +2\tau cos\gamma sin\gamma \right)$$

**Figure 11:**
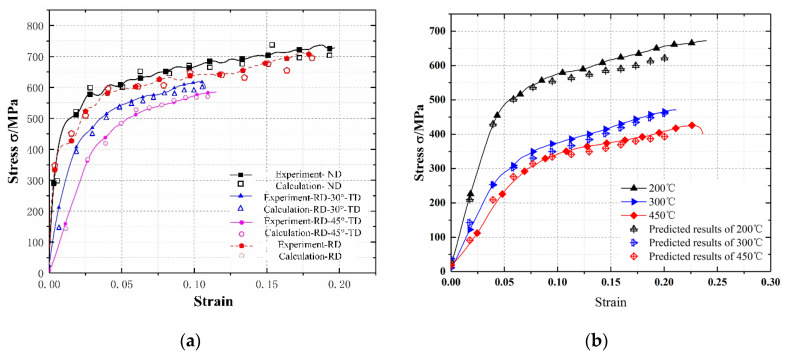
Stress–strain prediction curves under different loading conditions, (**a**) Predicted stress–strain curve at a strain rate of 0.2 × 10^4^ s^−^^1^. (**b**) Predicted stress–strain curves of the 45° specimens at different temperatures.

The stress–strain curves at arbitrary angles *γ*, *α* = 0, and *β* = 0 in three-dimensional space were predicted by combining Equations (18)–(20), and they are displayed in Figure 11. The accuracy of the constitutive equation containing the spatial angle was verified by predicting the strain curves at different temperatures and strain rates; the error was within 5%.

#### 4.3.4. Simulation Modeling of Dynamic Shock Compression Process

**Figure 12:**
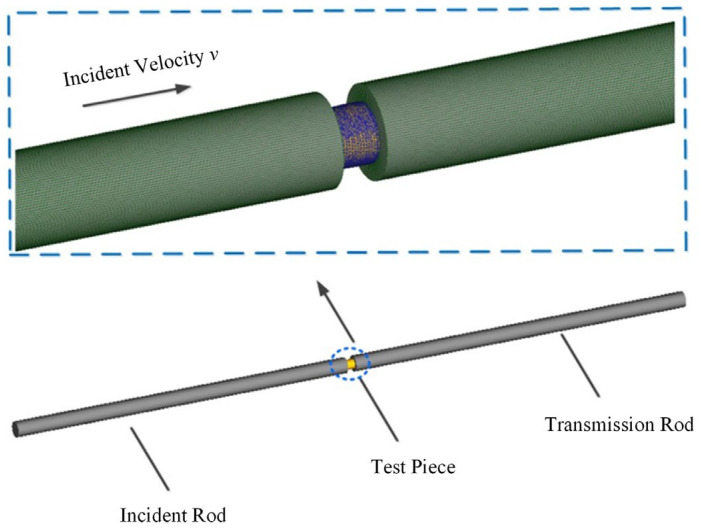
Schematic diagram of dynamic impact compression finite element model.

In order to further verify the accuracy of the constructed model, the deform module finite element geometric modeling is carried out according to the size and parameters of the SHPB test device. As shown in Figure 12, in order to reduce the amount of calculation of simulated deformation, the finite element model only retains the incident rod, the specimen and the transmission rod.

The finite element model does not consider the deformation of the device, and the incident rod and transmission rod are defined as rigid bodies. According to the parameters of the JC constitutive model and the material characteristic parameters of 7050-T7451, the mechanical response behavior of the specimen under load is set. Due to the complex changes of field variables in the material during dynamic impact compression, the tetrahedral constant strain element of DEFORM software is used to realize the adaptive meshing of regional field variables. Set the transmission rod to be fixed, and set the end surface of the test piece in contact with the transmission rod to be fixed. The initial incident velocity of the incident rod is set according to the impact rod velocity corresponding to the actual strain rate to strike the specimen. At the same time, the ambient temperature and heat conduction parameters of the specimen are set according to the material and temperature test parameters. Considering the calculation accuracy and efficiency, the friction factor is set to 0.4, the incremental steps are set to 10, and the analysis steps are set to 100.

**Figure 13:**
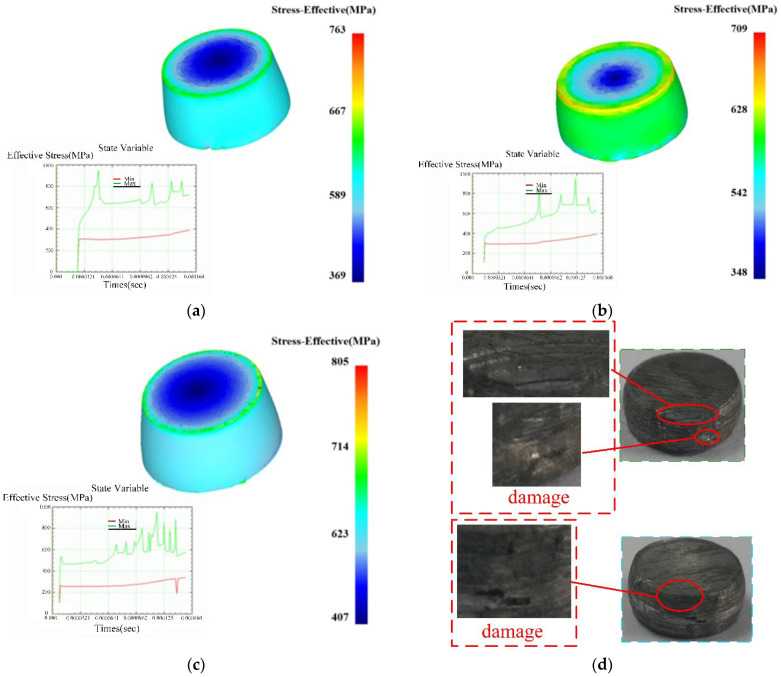
Comparison of simulated equivalent stress nephogram and damage forms of workpieces: (**a**) *ε* = 0.2, RD-30°-TD; (**b**) *ε* = 0.2, RD-45°-TD; (**c**) *ε* = 0.2, RD-60°-TD; (**d**) comparison diagram of sample damage.

Through the software post-processing module, the simulated data are processed and analyzed to obtain the changes of stress field, strain field and temperature field under different loading conditions. Figure 13 shows the equivalent stress nephogram of samples with different angles under the temperature of 200 °C and strain rate of 4000 s^−1^ when the strain is 0.2 during compression deformation. The results show that the maximum stress appears at the edge of the end face of the specimen, which is consistent with the edge spalling of the main failure form of the test specimen.

**Figure 14:**
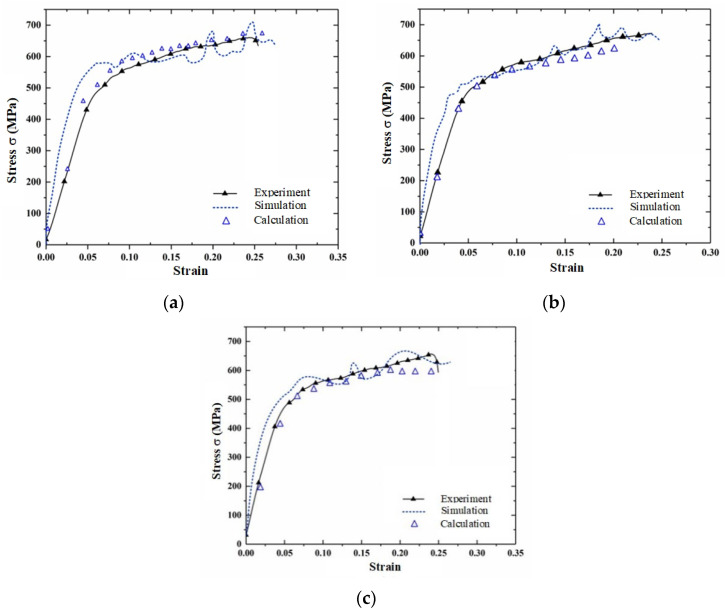
Comparison of test value, simulation value and model value of samples at different angles: (**a**) RD-30°-TD; (**b**) RD-45°-TD; (**c**) RD-60°-TD.

Figure 14 shows a comparison curve of test values, simulation values and calculated values of materials under different forming angles. By comparing the curves, it can be concluded that the maximum errors of the calculation and simulation results in the plastic deformation stage are 13.3%, 9.2%, and the maximum errors in the elastic stage are 16.6%. The constitutive relation mainly describes the plastic deformation behavior of materials under different loading conditions; thus, the prediction error in the elastic stage is large. However, the error is still within 20%. Therefore, considering the existence of uncontrollable errors in experimental measurement and simulation, the spatial modified constitutive model has high prediction accuracy. It can accurately describe the dynamic compression mechanical behavior of AA7050-T7451 under different forming angles in space.

## 5. Conclusions

In this work, theoretical expressions for *E*, *G*, and the stress σ, which can characterize the mechanical properties of the spatial and orthogonal coordinates at arbitrary angles *α*, *β*, and *γ*, were obtained and verified. The theoretical variations of *E*, *G* and σ with spatial angles *γ* were consistent with the experimental results. A coordinate transformation matrix [*A*] was obtained using projection relation and the theoretical expressions of σ between orthogonal and load coordinate systems. It was verified in the Results section that the stresses of TD, ND, RD under high strain rate and temperature can be accurately forecasted by the modified orthogonal constitutive model.

Combining the [*A*] with a modified orthogonal constitutive model, a spatial JC constitutive equation that includes the spatial angle *γ* was proposed for describing the dynamic mechanical properties of materials at different spatial angles (*γ*). The ratio between the theoretical and experimental stress under different loading conditions was within the 0.85–0.90 range because the influence of the preforming processes on the anisotropy was ignored. Therefore, the spatial JC constitutive equation was modified by introducing a correction factor *k* (0.86), and the results demonstrated and verified the accuracy of the theory and the method for characterizing the three-dimensional space anisotropy.

## Figures and Tables

**Table 1:** Correction values of the JC constitutive parameters *A*, *B*, *C*, and *n*.

Forming Directions	*A* (MPa)	*B* (MPa)	*C*	*n*
ND	365	516	0.042	0.26
TD	357	490	0.04	0.3
RD	342	452	0.029	0.26
